# Post-Capture Survival and Implications for By-Catch in a Multi-Species Coastal Gillnet Fishery

**DOI:** 10.1371/journal.pone.0166632

**Published:** 2016-11-18

**Authors:** Justin David Bell, Jeremy Martin Lyle

**Affiliations:** Institute for Marine and Antarctic Studies, University of Tasmania, Hobart, Tasmania, Australia; Department of Agriculture and Water Resources, AUSTRALIA

## Abstract

As fisheries shift towards ecosystem-based management, the need to reduce impacts on by-catch has been increasingly recognised. In this study the catch composition, discard rate, and post-capture survival of species caught by gillnets in Tasmania, Australia, was investigated. Over half the commercial gillnet catch was discarded, with discard rates of ~20% for target and >80% for non-target species. Capture condition, including initial mortality, was assessed using simple criteria for a range of species and related to soak duration. Delayed mortality was also assessed using tank trials and related to capture condition. By combining initial and delayed mortality rates post-capture survival was estimated. Longer soak durations generally resulted in slight, but significant, declines in capture condition and lower initial survival. Nonetheless, when combined with delayed survival, four of the five most commonly caught species (*Cheilodactylus spectabilis*, *Latridopsis forsteri*, *Aplodactylus arctidens*, *Cephaloscyllium laticeps*) exhibited high post-capture survival (83–100%) for soak durations within the maximum regulated range. Post-capture survival of the one remaining commonly caught species, *Notolabrus tetricus*, (typically discarded) declined with increased soak duration from 84% to 62%, suggesting that this species would benefit from a further reduction in maximum soak duration. Initial and delayed survival rates for the species retained for tank trials exhibited a significant linear relationship, which was used to estimate delayed survival rates for the rarer species. This method enabled the estimation of potential post-capture survival rates for a diverse range of species and may have application in other data-limited situations where the relationships between fishing practices and by-catch survival are uncertain. Overall, our results suggest that soak duration regulation has been effective in reducing by-catch mortality for many species, noting that some species have low survival rates regardless of soak duration.

## Introduction

The implementation of ecosystem-based fishery management and the need to restrict harvest levels have highlighted issues relating to by-catch and by-catch mortality [1]. By-catch represents that portion of the catch that is either discarded or released due to regulatory, economic or personal reasons [2]. Therefore, to fully assess the effects of fishing at individual population and ecosystem scales, it is necessary to quantify the levels of by-catch and associated rates of fishery induced mortality [3].

In terms of landed biomass, gillnetting is the fifth most productive fishing method globally [4], with fishery-specific by-catch rates ranging from 0–92% [5,6]. The overwhelming majority of research on gillnet by-catch to date has focussed on the impacts on seabirds, turtles and marine mammals (e.g. [7–11]), despite the majority of discards being fish.

In a major review of fishing mortality mitigation by entangling and entrapment fishing gears, only 11 out of 130 primary literature sources examined estimated post-capture survival of fish by-catch [12]. While we are aware of two additional studies relevant to the post-capture survival of fish taken by gillnetting [13,14], there remains a paucity of information on this issue. Most studies that have quantified post-capture survival of gillnet caught fish have focussed on single target species (e.g. [13,15–19]); very few have taken a multi-species approach to the broader impact of gillnet fisheries on by-catch survival [15,20,21].

The use of gillnets in Tasmania, Australia, commenced in the early 19^th^ century soon after European settlement [22], with both commercial and recreational sectors having access to this gear. The commercial fishery is a multi-species fishery with fishers adapting to species availability, market preferences and opportunities [23]. Commercial landings have declined since the late 1990s from >500 tonnes to ~100 tonnes per annum in recent years, with the number of active operators falling from around 250 to about 50 in the same period [24]. Historically, blue warehou (*Seriolella brama*), bastard trumpeter (*Latridopsis forsteri*), flounder (*Rhombosolea tapirina*) and yelloweye mullet (*Aldrichetta forsteri*) have been the main target species [24]. In the early 1990s, a fishery targeting banded morwong (*Cheilodactylus spectabilis*) for the domestic live fish trade developed and since the late 1990s commercial gillnet effort has been increasingly directed at this species. During this same period, the abundance of some key gillnet species, including blue warehou and bastard trumpeter, has declined [24]. Recreational gillnet fishers target many of these same species with two conspicuous differences: (i) banded morwong are rarely targeted or retained, and (ii) aquaculture escapees, mainly Atlantic salmon (*Salmo salar*), have become increasingly targeted by the recreational sector [25].

Following the introduction of minimum mesh sizes and no-netting areas in the 1890s, regulations governing the use of gillnets in Tasmania remained virtually unchanged up until the 1990s [22]. Since then a variety of regulations have been introduced including the licensing of recreational gillnets, net length restrictions for both sectors and the introduction of maximum permitted soak durations. The latter measure has been revised several times in an attempt to improve fishing practices, reduce wastage and minimise impacts on by-catch [24]. Overnight netting was banned in most Tasmanian waters in 2004 and in 2009 maximum permitted soak durations were introduced. A maximum soak duration of two hours currently applies in waters designated as shark refuge areas, while in state waters where overnight netting is banned a six-hour maximum soak duration applies.

An overall discard rate of over 35% (by number) has been reported for the Tasmanian recreational gillnet fishery [25], with discard rates highly variable between species. Several species are almost always discarded whereas for others, including target species, discarding occurs primarily due to regulatory reasons (size and bag limits) and as a result discard rates are relatively low. Discarding is known to occur in the commercial fishery and although rates have not been quantified, they are likely to be comparable to those for the recreational sector.

Due to the multi-species nature of gillnet catches and relatively high discard rates, the survival of by-catch is of interest and is the focus of the present study. In particular, we investigated how capture condition (physical condition, liveliness, evidence of barotrauma), immediate mortality (IM: mortality when the gear is retrieved) and delayed mortality (DM: mortalities occurring post release) are affected by gillnet soak duration since this represents the primary management measure that has been adopted to reduce impacts on by-catch and wastage. This information is not only relevant to an ongoing debate regarding the future of recreational gillnetting in Tasmania, often portrayed as an indiscriminate and wasteful fishing method, but also in understanding the broader impacts on inshore fish communities. This study proposes a simple approach to assessing the potential for by-catch survival based on capture condition that may have broader applicability to other small scale or data limited fisheries.

## Methods

### Field sampling

Research gillnetting (2362 gillnet deployments) was carried out between 2011 and 2013 using demersal gillnets of similar specifications to those used by recreational fishers (114 mm stretched mesh monofilament, 33 meshes deep and 50 m in length). Gillnets were deployed over inshore rocky reef and soft sediment habitats around Tasmania and typically hauled after 2 to 6 hours. Overnight gillnetting is only permitted in Macquarie Harbour, western Tasmania (42° 18' 56.52"S, 145° 23' 4.09"E), an area popular with recreational gillnetters [22]. To reflect this practice, research gillnets were also deployed overnight in Macquarie Harbour, with soak durations ranging between 12–19 hours. As the net was progressively retrieved on board the vessel, fish were removed from the net by either disentangling them or occasionally by cutting meshes. This reflected handling practices commonly used by recreational and commercial fishers (authors’ pers. obs.). Fish were identified, measured and a semi-quantitative assessment of vitality made based on liveliness and the extent of physical damage. Capture condition was assigned a five-point scale, with fish that were dead (unresponsive) upon retrieval of the net representing IM (Table 1). Fish were either released at the capture location, or were retained for post release survival experiments (detailed below).

**Table 1:** Capture condition categories assigned to gillnet caught fish.

Category	Vitality	Description
1	Excellent	Lively (vigorous body movement), no visible external injuries.
2	Good	Lively (vigorous body movement), minor physical damage: <5% scale loss, minor cuts and/or minor barotrauma.
3	Moderate	Alive (vigorous to moderately strong body movement) but with moderate physical damage: >5% scale loss, moderate cuts and/or moderate barotrauma.
4	Poor	Alive (moderate to weak body movement) but with limited responsiveness and/or extensive body damage (cuts, extensive scale loss), bleeding from the gills and/or severe barotrauma.
5	Dead	Unresponsive.

Commercial gillnet operations were also monitored during the study period (719 gillnet deployments) and comparable operational and catch information was recorded. Commercial gillnets ranged between 30 and 100 m in length, with mesh sizes of 114–140 mm. To identify the by-catch component, the fate of each fish, retained or discarded, was recorded along with its species, length and capture condition. Considerable care was taken to ensure that condition criteria were applied consistently throughout this study, with at least one experienced member of the research team present during all research and commercial catch sampling.

### Delayed mortality

Tank based survival trials involving gillnet caught fish were undertaken to examine the relationship between capture condition and DM. Land based trials, rather than sea cages, were used to monitor fish survival to avoid issues associated with exposure (swell) and the potential for fur seal interactions with sea cages.

Fish were captured while research gillnetting, removed from the net as described above and capture condition recorded. Fish were tagged (t-bar, Hallprint) or fin clipped to enable individuals to be re-identified, and then placed immediately into a 250 L, baffled and aerated tub of seawater on-board the capture vessel. The maximum stocking density in this tank was typically ~20 fish, although this was reduced if large individuals were retained. Gillnetting was conducted within close proximity of the Institute for Marine and Antarctic Studies (Hobart) to reduce transport times and, where possible, fish were selected to ensure that a range of capture condition categories was represented for each species. As a general rule, fish were held for <2 hours with water changes at ~1 hour intervals before transfer into 4000 L land based holding tanks. Flow through sea water with supplementary aeration was provided to each holding tank, providing ambient conditions of salinity, temperature and photoperiod during the experimental period. These were effectively identical to those the fish would have experienced had they been released where they were captured.

Fish were checked twice daily but not fed during a minimum four day holding period. Following the holding period, surviving fish were released near where they were captured. Mortalities were recorded and removed from the tanks. This period of time is commonly used in post-capture survival studies [12,13,26,27] since the majority of capture-induced mortalities tend to occur relatively soon after capture and longer holding periods risk introducing confounding factors arising from confinement.

### Data analysis

The effect of soak duration on capture condition (category) was explored by ordinal regression using the R package ‘MASS’. The relationship between soak duration and IM was assessed using binary logistic regression with season included as an independent variable. The year was divided into warm (November–April) and cool (May–October) water seasons to reflect the trend in water temperature (~10 m depth) within the study area [28].

The relationship between capture condition and DM was investigated using logistic regression, with pairwise comparisons made using Tukey’s contrasts with alpha levels corrected for multiple pair-wise comparisons [29]. Season (as defined above) was also included in this analysis as a factor where there was sufficient data in both seasons.

Post-capture survival, defined here as the proportion of the catch that survived capture (i.e. 1.0-IM) and at least three days post-capture observation (1.0-DM) was estimated using the combined research and commercial sampling dataset taking account of the relative proportion of each capture condition category by gillnet soak duration category. For this analysis soak durations were binned into the following categories: 1) 1.5–2.5 h ($\bar{\text{x}}$ = 1.96 ± 0.34 SD), 2) 2.5–3.5 h ($\bar{\text{x}}$ = 2.95 ± 0.26 SD), 3) 3.5–5 h ($\bar{\text{x}}$ = 4.07 ± 0.42 SD), 4) 5–8 h ($\bar{\text{x}}$ = 6.10 ± 0.53 SD), and 5) overnight ($\bar{\text{x}}$ = 16.19 ± 2.34 SD) to demonstrate the effect of soak duration on potential survival rates. Delayed survival was estimated directly from the tank trial results or, for those species for which DM relationships were not available, it was estimated using a linear model that related initial survival (regardless of soak duration) with delayed survival (regardless of capture condition) rates for those species for which at least 10 individuals had been retained for the tank trials. This latter approach assumes that the relationship between initial and delayed survival can be generalised as a proxy for post-capture survival in other fish species. Uncertainty associated with this approach was represented as 95% prediction intervals calculated for the estimates of delayed survival.

To increase the power of the above analyses, related species were grouped where they were morphologically similar and displayed similar patterns in capture condition and in particular IM rates. Species grouping was undertaken for flounders (*Ammotretis rostratus*, *Rhombosolea tapirina*), leatherjackets (family Monocanthidae–*Acanthaluteres vittiger*, *Eubalichthys gunnii*, *E*. *mosaicus*, *Meuschenia freycineti*, *M*. *australis*, *M*. *hippocrepis*, *M*. *scaber*, *M*. *flavolineata*, *M*. *venusta*) and stingarees (*Urolophus cruciatus*, *U*. *paucimaculatus*).

Statistical analysis and data manipulation was carried out with R version 3.0.1 [30] and the critical α-level for determining significance was 0.05.

### Ethical note

All sampling and experimentation was carried out in accordance with the Australian Code for the Care and Use of Animals for Scientific Purposes– 8th Edition 2013. The protocol was approved by the University of Tasmania Animal Ethics Committee (A0011313) and permits 10065, 11132 and 12238 issued by the Tasmanian Department of Primary Industries, Parks, Water and Environment under Section 14 of the Living Marine Resources Management Act 1995.

## Results

### Catch composition and discarding

A total of 70 fish species were recorded in the commercial gillnet catches with five species, namely banded morwong, bastard trumpeter, bluethroat wrasse (*Notolabrus tetricus*), draughtboard shark (*Cephaloscyllium laticeps*) and marblefish (*Aplodactylus arctidens*), accounting for 82% of the total catch by number (Table 2). These five species also accounted for 58% of the research gillnet catch and therefore represent the primary focus of this study. Sufficient data were available for a further 18 species to enable some limited analyses.

**Table 2:** Catch by number for commercial and research gillnet fishing, percent contribution of each species to the total catch and, for commercial catches, percentage of discards by species.

Common name	Scientific name	Commercial fishery				Research		Combined
		Catch (no.)	%	Discards (no.)	% discarded	Catch (no.)	%	Catch (no.)
Banded morwong	*Cheilodactylus spectabilis*	1644	42.8	365	22.2	686	12.0	2330
Marblefish	*Aplodactylus arctidens*	386	10.1	380	98.4	877	15.3	1263
Bluethroat wrasse	*Notolabrus tetricus*	404	10.5	382	94.6	803	14.1	1207
Draughtboard shark	*Cephaloscyllium laticeps*	525	13.7	518	98.7	474	8.3	999
Bastard trumpeter	*Latridopsis forsteri*	190	5.0	36	18.9	457	8.0	647
Spiny dogfish	*Squalus acanthias*	0	0.0	0	0.0	503	8.8	503
Elephantfish	*Callorhinchus milii*	3	0.1	1	33.3	310	5.4	313
Longsnout boarfish	*Pentaceropsis recurvirostris*	196	5.1	110	56.1	117	2.0	313
Leatherjacket	Monocanthidae	121	3.2	114	94.2	135	2.4	256
Australian salmon	*Arripis trutta*	48	1.3	1	2.1	174	3.0	222
Purple wrasse	*Notolabrus fucicola*	67	1.7	57	85.1	113	2.0	180
Maugean skate	*Zearaja maugeana*	0	0.0	0	0.0	180	3.2	180
Blue warehou	*Seriolella brama*	73	1.9	2	2.7	71	1.2	144
Magpie perch	*Cheilodactylus nigripes*	23	0.6	4	17.4	93	1.6	116
Blue grenadier	*Macruronus novaezelandiae*	0	0.0	0	0.0	115	2.0	115
Flounder	Rhombosoleinae	3	0.1	0	0	125	2.2	128
Herring cale	*Olisthops cyanomelas*	46	1.2	46	100	38	0.7	84
Atlantic salmon	*Salmo salar*	0	0.0	0	0.0	76	1.3	76
Jackass morwong	*Nemadactylus macropterus*	27	0.7	3	11.1	46	0.8	73
Red cod	*Pseudophycis bachus*	6	0.2	4	66.7	63	1.1	69
Gummy shark	*Mustelus antarcticus*	3	0.1	3	100	64	1.1	67
Melbourne skate	*Spiniraja whitleyi*	16	0.4	16	100	50	0.9	66
Stingaree	Urolophidae	9	0.2	9	100	54	0.9	63
Other		48	1.3	0	0.0	90	1.6	138
Total		3838		2195	54.9	5714		9552

Overall, just over half (by number) of the commercial catch was discarded (Table 2). Discard rates for key target species varied from ~20% for banded morwong and bastard trumpeter to over 90% for non-target species such as marblefish, bluethroat wrasse and draughboard shark. Very high discard rates were also evident for several of the minor species, including leatherjackets, purple wrasse (*Notolabrus fucicola*) and herring cale (*Odax cyanomelas*) (Table 2).

In terms of catch composition, the main difference between the commercial and research samples was the representation of spiny dogfish (*Squalus acanthias*), elephantfish (*Callorhinchus milii*), Maugean skate (*Zearaja maugeana*), blue grenadier (*Macruronus novaezelandiae*), flounder and Atlantic salmon. Collectively these species accounted for 23% of total numbers in the research sample compared with <0.2% in the commercial catch sample. This difference was due to the greater emphasis of research fishing in sheltered waters and over soft sediment habitats (including Macquarie Harbour), undertaken to reflect recreational targeting of escapee salmonids.

### Capture condition

Capture condition declined (i.e. condition category increased) as soak duration increased in 20 of the 23 species investigated (Table 3), though this relationship was not always significant. Most of the species for which condition was not significantly affected by soak duration can be grouped into two categories; those resilient to external damage and typically in excellent or good condition (category 1 or 2) irrespective of soak duration, such as draughtboard shark, leatherjackets and Melbourne skate (*Spiniraja whitleyi*), and those that tended to be in poor condition or dead (category 4 or 5) irrespective of soak duration, such as herring cale, Australian salmon (*Arripis trutta*) and blue grenadier.

**Table 3:** Ordinal regression investigating the effect of gillnet soak duration on capture condition.

Species	Estimate	Std. error	T value	*p*
Atlantic salmon	0.008	0.006	1.297	0.195
Australian salmon	0.068	0.056	1.212	0.226
Banded morwong	0.166	0.025	6.682	<0.001
Bastard trumpeter	0.170	0.037	4.609	<0.001
Bluethroat wrasse	0.056	0.029	1.903	0.057
Blue grenadier	0.318	0.214	1.488	0.137
Blue warehou	0.081	0.039	2.089	0.037
Draughtboard shark	-0.006	0.005	-1.137	0.256
Elephantfish	0.139	0.034	4.049	<0.001
Flounders	0.012	0.029	0.413	0.680
Gummy shark	0.184	0.233	0.791	0.429
Herring cale	0.145	0.142	1.020	0.308
Jackass morwong	0.243	0.140	1.731	0.083
Leatherjackets	0.072	0.120	0.597	0.551
Longsnout boarfish	0.115	0.086	1.334	0.182
Magpie perch	0.213	0.109	1.945	0.052
Marblefish	0.143	0.024	5.863	<0.001
Maugean skate	0.158	0.027	5.767	<0.001
Melbourne skate	-0.345	0.305	-1.133	0.257
Purple wrasse	0.130	0.076	1.716	0.086
Red cod	0.190	0.044	4.348	<0.001
Stingarees	-0.160	0.171	-0.936	0.349
Spiny dogfish	0.044	0.010	4.614	<0.001

### Immediate mortality

Of the five most common gillnet species, the rate of IM for only two, bluethroat wrasse and marblefish, increased with soak duration and was higher during the warm season (Table 4). In contrast, the relationships between IM and soak duration and season for banded morwong and bastard trumpeter were not significant; both species experienced low IM (generally <5%) irrespective of soak duration or season. The absence of any instances of IM for draughtboard shark precluded modelling.

**Table 4:** Logistic regression of variation in immediate mortality (IM) rate due to variation in soak duration and sampling season (refer text). Draughtboard shark, Melbourne skate and stingarees could not be included in this analysis as these species experienced no instances of IM.

Species	Coefficient	Estimate	Std. error	Z value	*p*
Atlantic salmon	Soak duration	-0.123	0.053	-2.328	0.020
	Season	-0.891	0.553	-1.608	0.108
Australian salmon	Soak duration	-0.321	0.110	-2.913	0.003
	Season	0.761	0.184	4.142	<0.001
Banded morwong	Soak duration	-0.166	0.132	-1.259	0.208
	Season	0.595	0.371	1.603	0.109
Bastard trumpeter	Soak duration	-0.082	0.128	-0.644	0.520
	Season	0.411	0.277	1.484	0.138
Bluethroat wrasse	Soak duration	-0.410	0.048	-8.460	<0.001
	Season	0.351	0.082	4.284	<0.001
Blue grenadier*	Soak duration	-0.319	0.214	-1.488	0.137
Blue warehou	Soak duration	-0.076	0.041	-1.862	0.063.
	Season	0.997	0.210	4.755	<0.001
Elephantfish	Soak duration	-0.136	0.051	-2.699	0.007
	Season	-0.406	0.273	-1.489	0.136
Flounder	Soak duration	-0.027	0.075	-0.357	0.721
	Season	-0.134	0.719	-0.187	0.852
Gummy shark	Soak duration	-0.382	0.264	-1.448	0.148
	Season	-0.108	0.374	-0.288	0.773
Herring cale	Soak duration	-0.364	0.160	-2.280	0.023
	Season	0.534	0.241	2.215	0.027
Jackass morwong	Soak duration	-0.684	0.202	-3.380	<0.001
	Season	0.177	0.302	0.584	0.559
Leatherjacket	Soak duration	-0.070	0.171	-0.406	0.685
	Season	-0.017	0.462	-0.037	0.971
Longsnout boarfish*	Soak duration	-1.640	1.439	-1.140	0.254
Magpie perch	Soak duration	0.077	0.271	0.283	0.777
	Season	0.090	0.445	0.202	0.840
Marblefish	Soak duration	-0.289	0.105	-2.762	0.005
	Season	0.560	0.270	2.072	0.038
Maugean skate*	Soak duration	-0.487	0.152	-3.200	0.001
Purple wrasse	Soak duration	-0.276	0.180	-1.534	0.125
	Season	0.338	0.327	1.034	0.301
Red cod	Soak duration	-0.207	0.046	-4.547	<0.001
	Season	0.896	0.574	1.560	0.119
Spiny dogfish	Soak duration	-0.086	0.024	-3.583	<0.001
	Season	0.151	0.260	0.582	0.561

* insufficient data to include season as a factor.

Amongst the less frequently encountered species, longer soak durations resulted in significantly higher IM for eight species, and season was a significant factor in just three instances (Table 4). Very low rates of IM for a further five species meant that the relationships between IM and soak duration and/or season were not significant, whereas the lack of significance in the relationship for blue grenadier was due to very high IM regardless of soak duration. There were no instances of IM observed for Melbourne skate or the stingarees.

### Delayed mortality

A total 36 species were retained for tank survival experiments, however, only ten of these were represented by sample sizes of >10 individuals (Table 5). For eight of these species there was a total of 38 mortalities within the four day holding period: 76.3% within 24 h, 92.1% by day 2 and 100% by day 3. By contrast, two species (elephantfish and Australian salmon) had a total of 17 mortalities, 41.2% occurring within 24 h, 52.9% by day 3 and 47.1% on day 4. This unusual pattern of mortalities suggests that there may have been confounding factors not directly related to capture stress for these two species. Previous experience holding elephantfish in captivity [31] indicated that the species does not survive for long periods of time in tanks of sizes similar to those used in the present study while Australian salmon are a semi-pelagic species and did not appear to adapt well to captivity. As such, three days was determined to be an appropriate time period for estimating short-term delayed mortality for most species; a longer holding period risked introducing confounding factors associated with confinement.

**Table 5:** Numbers and proportion by condition category that survived during the delayed mortality trials (three days, post capture).

Species	Category 1			Category 2			Category 3			Category 4		
	No.	No. survived	Proportion survived	No.	No. survived	Proportion survived	No.	No. survived	Proportion survived	No.	No. survived	Proportion survived
Bastard trumpeter	3	3	1.00	37	36	0.97	55	49	0.89	34	27	0.79
Banded morwong	22	22	1.00	87	85	0.98	14	14	1.00	5	4	0.80
Bluethroat wrasse	26	26	1.00	38	36	0.95	43	38	0.88	19	10	0.53
Marblefish	18	18	1.00	36	35	0.97	11	11	1.00	16	14	0.88
Draughtboard shark	39	39	1.00	32	32	1.00	0	0	-	0	0	-
Australian salmon	5	5	1.00	19	18	0.95	3	1	0.33	4	2	0.50
Elephantfish	4	4	1.00	20	18	0.90	5	4	0.80	1	0	0.00
Leatherjacket	13	13	1.00	15	15	1.00	1	0	0.00	0	0	-
Magpie perch	4	4	1.00	16	15	0.94	2	2	1.00	0	0	-
Longsnout boarfish	2	2	1.00	10	10	1.00	1	1	1.00	0	0	-

Season was not a significant factor in DM rates for any of the five main gillnet species and was therefore removed as a factor in subsequent analyses. Since none of the draughtboard shark retained for survival trials died during the holding period it was not possible to undertake modelling for this species. Banded morwong were robust, with the majority of condition category 1 and 2 individuals surviving the holding period. Fish in moderate and poor condition were rare in both research and commercial catches and thus categories 3 and 4 were represented by few individuals in the survival trials (Table 5). The very low DM rate meant the logistic regression model did not initially fit the data, however, when categories 1 to 3 were combined the model did perform adequately, indicating a significant increase in DM for condition category 4 (Table 6).

**Table 6:** Logistic regression of survival probability by condition category. In all four species, the best model involved the comparison of condition categories 1:3 combined with category 4 (detailed in text).

Species	Condition	Estimate	Std. error	Z value	*p*
Banded morwong	(Intercept)	4.103	0.713	5.755	<0.001
	1:3–4	-2.716	1.326	-2.049	0.041
Bastard trumpeter	(Intercept)	2.531	0.393	6.446	<0.001
	1:3–4	-1.182	0.578	-2.044	0.041
Bluethroat wrasse	(Intercept)	2.659	0.391	6.802	<0.001
	1:3–4	-2.554	0.603	-4.233	<0.001
Marblefish	(Intercept)	4.159	1.008	4.127	<0.001
	1:3–4	-2.213	1.260	-1.757	0.079

Marblefish were also resilient to gillnet capture. Nil or minimal DM for all but fish in poor condition meant that the logistic model fitted the data poorly and there was no significant relationship between DM and condition, even when categories 1–3 were combined (Table 6). Interestingly, the majority (88%) of the condition 4 fish survived the tank trial period despite many bleeding from the gills at capture. Each appeared to have recovered from their injuries within the holding period.

Bastard trumpeter typically sustained scale loss and bruising from contact with the gillnet meshes and thus fish in excellent condition were uncommon (Table 5). All category 1 fish survived the holding period and there was only a single category 2 mortality, thus condition categories 1–3 were combined for modelling. There was a significant difference between the DM rates for categories 1–3 combined and that for category 4 fish (Table 6).

Bluethroat wrasse was the only common gillnet species regularly encountered in moderate to poor condition and the only species to exhibit relatively high DM rates, which increased from 0% for condition category 1 to 90% for category 4 fish (Table 7). Although the logistic model fitted the data with all condition categories included individually, the absence of mortalities in category 1 prevented this condition being used in pairwise comparisons. Combining categories 1 and 2 improved the model fit, but there was no differential rate of mortality between 1 and 2 combined and category 3 (Tukey’s test, *p* = 0.230). As such, categories 1–3 were combined and this combined category had a significantly higher survival probability than did category 4 (Table 6).

**Table 7:** Initial survival, delayed survival (proportion of initial survival) and estimated post-capture survival by soak duration category.

		Survival rate (proportion)		
Species	Soak duration	Initial	Delayed	Total
Atlantic salmon	1	0.95	0.94 (0.84–1.04)	0.89 (0.80–0.99)
	2	0.60	0.79 (0.65–0.93)	0.47 (0.39–0.56)
	3	0.60	0.79 (0.65–0.93)	0.47 (0.39–0.56)
	4	0.50	0.74 (0.58–0.90)	0.37 (0.29–0.45)
	5	0.41	0.70 (0.52–0.89)	0.29 (0.21–0.36)
Australian salmon	1	0.70	0.83 (0.71–0.95)	0.58 (0.50–0.67)
	2	0.81	0.88 (0.78–0.98)	0.71 (0.63–0.80)
	3	0.44	0.72 (0.54–0.89)	0.32 (0.24–0.39)
	4	0.42	0.71 (0.53–0.89)	0.30 (0.22–0.37)
Banded morwong*	1	0.99	0.98	0.97
	2	0.99	0.99	0.98
	3	0.99	0.99	0.98
	4	0.99	0.98	0.97
Bastard trumpeter*	1	0.97	0.91	0.89
	2	0.94	0.93	0.87
	3	1.00	0.95	0.95
	4	0.95	0.89	0.84
Blue grenadier	1	0.00	0.00	0.00
	2	0.00	0.00	0.00
	3	0.06	0.54 (0.27–0.83)	0.03 (0.02–0.05)
	4	0.20	0.61 (0.37–0.85)	0.12 (0.07–0.17)
	5	0.00	0.00	0.00
Blue warehou	1	0.67	0.82 (0.69–0.94)	0.55 (0.46–0.63)
	2	0.69	0.83 (0.71–0.95)	0.57 (0.49–0.65)
	3	0.21	0.62 (0.38–0.85)	0.13 (0.08–0.18)
	4	0.42	0.71 (0.53–0.89)	0.30 (0.22–0.37)
	5	0.47	0.73 (0.56–0.90)	0.34 (0.26–0.42)
Bluethroat wrasse*	1	0.92	0.91	0.84
	2	0.91	0.9	0.82
	3	0.81	0.9	0.73
	4	0.69	0.9	0.62
Draughtboard shark*	1	1.00	1.00	1.00
	2	1.00	1.00	1.00
	3	1.00	1.00	1.00
	4	1.00	1.00	1.00
Elephantfish	1	0.97	0.95 (0.85–1.05)	0.92 (0.83–1.02)
	2	0.93	0.93 (0.84–1.03)	0.87 (0.78–0.96)
	3	0.94	0.94 (0.84–1.04)	0.88 (0.79–0.97)
	4	0.92	0.93 (0.83–1.03)	0.85 (0.76–0.94)
	5	0.80	0.88 (0.77–0.98)	0.70 (0.62–0.78)
Flounder	1	0.95	0.94 (0.84–1.04)	0.89 (0.80–0.99)
	2	1.00	0.96 (0.86–1.06)	0.96 (0.86–1.06)
	3	1.00	0.96 (0.86–1.06)	0.96 (0.86–1.06)
	4	1.00	0.96 (0.86–1.06)	0.96 (0.86–1.06)
	5	0.95	0.94 (0.84–1.04)	0.90 (0.80–0.99)
Gummy shark	1	0.82	0.89 (0.78–0.99)	0.73 (0.64–0.81)
	2	0.74	0.85 (0.74–0.96)	0.63 (0.54–0.71)
Herring cale	1	0.78	0.87 (0.76–0.97)	0.68 (0.59–0.76)
	2	0.76	0.86 (0.75–0.97)	0.65 (0.57–0.74)
	3	0.61	0.79 (0.65–0.93)	0.48 (0.40–0.57)
	4	0.43	0.71 (0.65–0.93)	0.31 (0.23–0.38)
Jackass morwong	1	0.92	0.93 (0.83–1.03)	0.85 (0.76–0.94)
	2	0.89	0.92 (0.82–1.01)	0.82 (0.73–0.09)
	3	0.33	0.67 (0.46–0.87)	0.22 (0.15–0.29)
	4	0.44	0.72 (0.54–0.89)	0.32 (0.24–0.39)
Leatherjacket	1	1.00	0.96 (0.86–1.06)	0.96 (0.86–1.06)
	2	1.00	0.96 (0.86–1.06)	0.96 (0.86–1.06)
	3	0.99	0.96 (0.86–1.06)	0.94 (0.85–1.05)
	4	0.99	0.96 (0.86–1.06)	0.95 (0.85–1.05)
Longsnout boarfish	1	1.00	0.96 (0.86–1.06)	0.96 (0.86–1.06)
	2	1.00	0.96 (0.86–1.06)	0.96 (0.86–1.06)
	3	1.00	0.96 (0.86–1.06)	0.96 (0.86–1.06)
	4	0.97	0.95 (0.85–1.05)	0.92 (0.83–1.02)
Magpie perch	1	0.96	0.95 (0.85–1.04)	0.91 (0.81–1.00)
	2	0.91	0.92 (0.83–1.02)	0.84 (0.75–0.93)
	3	1.00	0.96 (0.86–1.06)	0.96 (0.86–1.06)
	4	0.97	0.95 (0.85–1.05)	0.92 (0.83–1.02)
Marblefish*	1	0.99	0.98	0.97
	2	0.98	0.98	0.96
	3	0.97	0.98	0.95
	4	0.96	0.97	0.93
Maugean skate	1	1.00	0.96 (0.86–1.06)	0.96 (0.86–1.06)
	2	1.00	0.96 (0.86–1.06)	0.96 (0.86–1.06)
	3	1.00	0.96 (0.86–1.06)	0.96 (0.86–1.06)
	4	1.00	0.96 (0.86–1.06)	0.96 (0.86–1.06)
	5	0.91	0.92 (0.83–1.02)	0.84 (0.75–0.93)
Melbourne skate	1	1.00	0.96 (0.86–1.06)	0.96 (0.86–1.06)
	2	1.00	0.96 (0.86–1.06)	0.96 (0.86–1.06)
	3	1.00	0.96 (0.86–1.06)	0.96 (0.86–1.06)
	4	1.00	0.96 (0.86–1.06)	0.96 (0.86–1.06)
Purple wrasse	1	0.96	0.95 (0.86–1.06)	0.92 (0.81–1.00)
	2	0.98	0.96 (0.86–1.06)	0.94 (0.84–1.03)
	3	0.87	0.91 (0.81–1.01)	0.79 (0.70–0.88)
	4	0.91	0.92 (0.83–1.02)	0.84 (0.75–0.93)
Red cod	1	0.81	0.88 (0.78–0.98)	0.71 (0.63–0.80)
	2	0.80	0.88 (0.77–0.98)	0.70 (0.62–0.78)
	3	0.50	0.74 (0.58–0.90)	0.37 (0.29–0.45)
	4	0.00	0.00	0.00
	5	0.10	0.57 (0.30–0.83)	0.06 (0.03–0.08)
Spiny dogfish	1	0.92	0.93 (0.83–1.03)	0.85 (0.76–0.94)
	2	0.93	0.93 (0.84–1.03)	0.87 (0.78–0.96)
	3	0.94	0.94 (0.84–1.04)	0.88 (0.79–0.97)
	4	0.94	0.94 (0.84–1.04)	0.88 (0.79–0.97)
	5	0.82	0.89 (0.78–0.99)	0.73 (0.64–0.81)
Stingarees	1	1.00	0.96 (0.86–1.06)	0.96 (0.86–1.06)
	2	1.00	0.96 (0.86–1.06)	0.96 (0.86–1.06)
	3	1.00	0.96 (0.86–1.06)	0.96 (0.86–1.06)
	4	1.00	0.96 (0.86–1.06)	0.96 (0.86–1.06)

**Fig 1:**
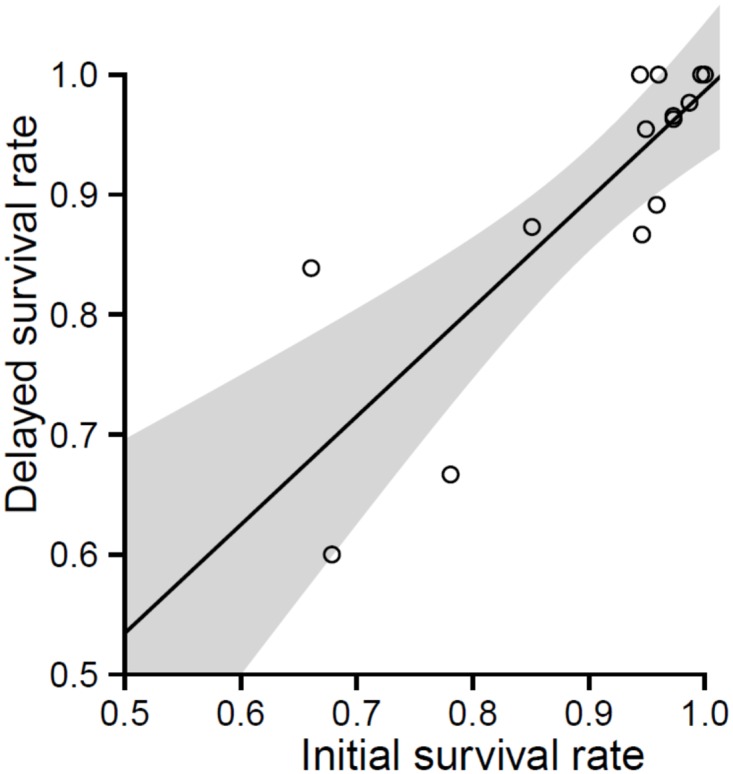
Linear relationship between initial survival rate and delayed survival rate. Shaded area represents 95% confidence interval.

*indicates species for which delayed survival was based on tank trial results, delayed survival for all other species was estimated using the model in Fig 1 (95% prediction intervals provided in parentheses). Delayed survival was not estimated when initial survival was zero. N is the number of individuals captured within each soak duration.

There was no DM of longsnout boarfish during the holding period (Table 5). Of the other species retained for the tank trials, DM rates were below 5% for leatherjackets and magpie perch (*Cheilodactylus nigripes*) and were higher, at around 15% for Australian salmon and elephantfish. As noted above, however, there is greater uncertainty associated with the reliability of the results for these latter species due to the potentially confounding factors arising from confinement.

### Post-capture survival

Post-capture survival rates for the five major gillnet species have been estimated by taking into account the relationships between capture condition and soak duration in the combined research and commercial datasets and the effect of capture condition on subsequent survival (Table 7). This approach indicated that the post-capture survival rate for draughtboard shark was effectively 100% within the range of soak durations tested. The overall post-capture survival was very high for banded morwong (~97%), with little variation associated with soak duration. Marblefish were also assessed to have high post-capture survival although the rate did decline slightly, from 97 to 93%, as soak duration increased. Estimated post-capture survival rates for bastard trumpeter were slightly lower and more variable, ranging between 95 and 84%, the lowest survival associated with the longest soak durations. Delayed rather than immediate mortality had greater effect in influencing post-capture survival for this species. For bluethroat wrasse, post-capture survival declined from 84 to 62% as soak duration increased, driven mainly by the increase in IM rather than DM.

The relationship between initial survival (IS) and delayed survival (DS) for those species where DM was estimated experimentally was used as a proxy for DS for the less common species, where:

$$DS=0.904\;\left(IS\right)+0.082;p=<0.001,\;R^{2}=0.685$$

(Fig 1)

This analysis suggested that in addition to banded morwong, marblefish and bastard trumpeter, several other reef associated species, namely longsnout boarfish, leatherjackets, magpie perch and purple wrasse, also had the potential for high post-capture survival (>90%) within the range of soak durations examined (Table 7). There were, however, some exceptions, they included jackass morwong, herring cale and red cod, which exhibited poor survival (<50%), especially in the longer soak durations. As a group, the semi-pelagic species such as Atlantic salmon, Australian salmon, blue warehou and blue grenadier tended to exhibit low post-capture survival that declined markedly, to <50% in the longest soak durations. Estimated post-capture survival rates for the chondrichthyan species were variable, with the Batoids (Maugean skate, Melbourne skate, stingarees) tending to have high survival rates (>90%) whereas the sharks (gummy shark, spiny dogfish) and holocephalan (elephantfish) exhibited moderate post-capture survival (~60–90%).

## Discussion

The present study represents one of very few (e.g. [15,20,21]) to take a multi-species approach to assessing post-capture survival in gillnet fisheries. Although small scale, the Tasmanian fishery is complex as it comprises both commercial and recreational sectors and targets a diverse range of species in a variety of coastal habitats. The study has demonstrated that the maximum gillnet soak duration regulations implemented as a strategy to improve fishing practices appear to be effective for most, but not all, species in facilitating high post-capture survival, thereby reducing wastage and the ecosystem impacts of the fishery.

Discarding in the Tasmanian commercial gillnet fishery was determined to represent just over half of the catch based on numbers and compares with a discard rate of 35% for the recreational gillnet fishery [25]. Discard rates varied markedly between species, with both commercial and recreational sectors discarding the vast majority (generally greater than 80%) of non-target species. By contrast, discard rates for target species, such as bastard trumpeter and banded morwong tended to be much lower (generally less than 20%). Discarding of non-target species was mainly due to undesirability for consumption (recreational fishers) [25] or lack of markets (commercial fishers), whereas discarding of target species was mainly linked to regulation, in particular bag or size limits (authors’ pers. obs., [25]).

For target and by-product species with relatively low discard rates, accounting for and reducing post-capture mortality is important in assessing and managing the impacts of fishing where regulatory provisions necessitate that some catch will be discarded. For the non-target species that experience high discard rates, fishing practices that facilitate high post-capture survival will have obvious benefits in reducing the impacts on these species and the broader ecosystem.

Reviews of by-catch (discard) mortality from gillnets indicate that it is highly variable and species specific, ranging from 0–100% [26,32], with a reported mean of about 40% across the range of species for which information is available [12]. Mortality rates tend to increase with longer soak durations, poor handling, selectivity toward juveniles (size is often a significant factor), greater capture depths, elevated water/air temperatures and predation/envenomation [5,12,15,20,21,26,29,33–35]. However, due in part to progressive changes to the management of gillnetting in Tasmania, in particular the introduction of maximum permitted soak durations, coupled with the small scale of operations, many of these factors do not appear to be especially problematic for the Tasmanian fishery. In fact, our findings are generally supportive of Broadhurst and colleagues [15] who suggest that by-catch mortality in small scale, non-industrial, gillnet fisheries tends to be reduced due to typically short soak durations, smaller catches and reduced sorting and handling times. In such situations, post-capture survival can often exceed 90% [13,15,32]. Consistent with this generalisation, post-capture survival rates estimated for many of the study species were high (> 85%) based on the current 6-h maximum soak duration, implying that further reductions in the maximum permitted soak duration would be unlikely to have substantial additional benefit in reducing by-catch mortality. A reduction in maximum soak duration to about 3 h would, however, reduce the mortality of several species including bluethroat wrasse, purple wrasse, herring cale, red cod, and Australian salmon. In contrast, the generally high mortality of gillnet caught blue warehou and blue grenadier regardless of soak duration suggests that any reductions in soak durations would be of marginal benefit for these species, noting the former is a target species that is rarely discarded. Nonetheless, when it is considered that over three quarters of recreational gillnet sets during the late 1990s involved overnight deployments, and more than a quarter of these were deployed for periods of about 24 h [36], it is likely that recent management initiatives have contributed to a substantial reduction in overall by-catch mortality in the Tasmanian gillnet fishery.

Studies estimating post-capture survival often involve some form of captive observation, typically holding fish in tanks or sea cages. A key consideration of this approach is that it can introduce or remove sources of mortality [37]. For instance, holding of fish in enclosures can eliminate the potential for predation after release but may introduce confounding effects due to additional handling, transport and confinement. These latter factors can induce additional stress [38], and potentially captive-related mortality, in addition to any capture related effects. The inclusion of controls is, therefore, recommended to correct for captivity-related mortality when estimating survival rates [12]. This typically involves capturing fish using alternative techniques; however, in the present study, with the exception of bluethroat wrasse, none of the common gillnet species are vulnerable to other fishing gears. Nevertheless, the fact that all condition category 1 and virtually all category 2 fish survived the four day holding period implies that confounding factors affecting the survival response were likely to have been minimal for most of the study species. Australian salmon and elephantfish were exceptions and thus there is greater uncertainty surrounding inferences about the post-capture survival potential for these species.

The condition index utilised in this study proved to be a relatively robust predictor of delayed mortality for many species, with mortalities most common amongst individuals in the poorest condition (category 4). In practice, because of the low rates of delayed mortality associated with condition categories 1–3, it was necessary to combine them when modelling the effects of condition on delayed mortality. This suggests that it would be feasible to reduce the condition categories to a 3 or 4 tiered system which would further streamline the assessment process, avoiding more complicated schemes of vitality assessment that can be challenging to implement during on board observation of fishing operations. Furthermore, by applying the relationship between initial and delayed survival determined for the more common species, it was possible to estimate the post-capture survival potential for the less commonly caught species. In data limited situations, such an approach could be utilised within an ecological risk assessment framework to assess the likely impacts of gillnetting on by-catch, including the relative effects of varying fishing practices (such as soak durations) on post-capture survival. The broader applicability of this approach does, however, require validation on a greater number of species and, in particular, on species subject to high rates of IM as these were rare in the present study.

Although the implementation of maximum soak duration regulations represents a positive step towards reducing by-catch mortality, the Tasmanian fishery does interact with a variety of threatened, endangered and protected species [25,39,40], including the endangered Maugean skate. This species has a very limited distribution encompassing just two west coast Tasmanian estuaries [41], one of which is a no-take marine reserve (Bathurst Harbour) and the other is the only region where overnight gillnetting is permitted (Macquarie Harbour). In the present study, while there were no Maugean skate mortalities recorded in the daytime sets, mortalities did occur in some overnight sets, suggesting that post-capture survival could be as low as 85% for these longer deployments. Closures of key areas of Maugean skate habitat to gillnetting were implemented in 2015 to reduce the probability of skate by-catch. The effectiveness of these measures in reducing interactions and incidental mortality, noting that overnight netting is still permitted, have yet to be assessed. While considerable progress has been made in improving fishing practices and reducing by-catch mortality, managing interactions with protected species as well as general issues associated with multi-species fisheries assessment remain important challenges for the sustainable management of the Tasmanian gillnet fishery.
